# Predictors of Lifestyle Intervention Attrition or Weight Loss Success in Women with Polycystic Ovary Syndrome Who Are Overweight or Obese

**DOI:** 10.3390/nu11030492

**Published:** 2019-02-26

**Authors:** Lisa J. Moran, Manny Noakes, Peter Clifton, Jon Buckley, Grant Brinkworth, Rebecca Thomson, Robert J. Norman

**Affiliations:** 1 School of Medicine, Robinson Research Institute, University of Adelaide, Adelaide, SA 5000, Australia; robert.norman@adelaide.edu.au; 2 Monash Centre for Health Research and Implementation, School of Public Health and Preventive Medicine, Monash University, Melbourne, Vic 3163, Australia; 3 CSIRO Division of Health Sciences and Nutrition, Australia; manny.noakes@csiro.au (M.N.); grant.brinkworth@csiro.au (G.B.); 4 Alliance for Research in Exercise, Nutrition and Activity (ARENA), Sansom Institute for Health Research, University of South Australia, Adelaide, SA 5000, Australia; peter.clifton@unisa.edu.au (P.C.); jon.buckley@unisa.edu.au (J.B.); r.thomson@adelaide.edu.au (R.T.); 5 Fertility SA, Adelaide, SA 5000, Australia

**Keywords:** weight loss, attrition, polycystic ovary syndrome, diet, exercise, lifestyle

## Abstract

**Background/objectives:** Polycystic ovary syndrome (PCOS) is a common condition in reproductive-aged women. Weight management is a first-line treatment for PCOS according to international evidence-based guidelines. However, the factors associated with attrition or success in weight loss interventions are not known for women with PCOS. The objective of this study was to identify characteristics associated with attrition and weight loss success in women with PCOS and overweight or obesity undergoing weight loss interventions. **Methods:** Four randomised controlled clinical weight loss trials comprising energy restricted diets and/or exercise interventions of 2–8 months duration. The interventions were conducted over 2001–2007 in outpatient clinical research centres with *n* = 221 premenopausal women with PCOS and overweight/obesity recruited through community advertisement. The main outcome measures were attrition and ≥5% weight loss at 2 months and study completion. **Results:** Weight loss was 5.7 ± 2.9 kg at 2 months and 7.4 ± 5.3 kg after study completion (*p* < 0.001). Attrition was 47.1% and ≥5% weight loss occurred in 62.5% and 62.7% of women at 2 months and study completion respectively. Baseline depressive symptoms (OR 1.07 95% CI 0.88, 0.96, *p* = 0.032) and lower appointment attendance by 2 months (OR 0.92 95% CI 0.88, 0.96, *p* < 0.001) were independently associated with attrition. Lower appointment attendance over the whole study was independently associated with not achieving ≥5% weight loss at study completion (OR 0.95 95% CI 0.92, 0.99, *p* = 0.020). **Conclusions:** Despite high attrition, successful weight loss was achieved by 63% of women with PCOS in a clinical research setting. Higher baseline depressive symptoms were associated with greater attrition and higher appointment attendance was associated with lower attrition and greater weight loss success. These finding have implications for development of successful weight management programs in PCOS.

## 1. Introduction

**Clinical trial registration:** Three of the studies referred to in this article were conducted prior to mandatory clinical trial registration [1,2,3] and the remaining study was registered with the Australian Clinical Trial Registry (ACTRN12606000198527) [4].

Polycystic ovary syndrome (PCOS) is a common condition affecting up to 18% of reproductive-aged women [5]. According to the European Society of Human Reproduction and Embryology/American Society for Reproductive Medicine (ESHRE/ASRM) criteria it is diagnosed according to 2 of the 3 features of ovulatory disturbance, hyperandrogenism or polycystic ovaries with exclusion of other factors [6]. Women can present with reproductive (infertility and pregnancy complications), metabolic (increased risk factors for and potential higher prevalence of impaired glucose tolerance, type 2 diabetes and cardiovascular disease) and psychological (worsened quality of life and increased anxiety and depression) disturbances [7,8,9,10]. Insulin resistance is proposed as a key aetiological feature which is present even in lean women with PCOS in an intrinsic form mechanistically distinct to that associated with obesity [11]. Where weight gain occurs in PCOS, this is compounded by obesity-associated insulin resistance. Furthermore, there is a bidirectional relationship between PCOS and excess weight whereby women with PCOS may also have an increased prevalence of longitudinal weight gain [12] and overweight and obesity [13]. 

Correspondingly, overweight and obesity are key features to consider in PCOS management. Evidence-based guidelines recommend weight management (prevention of weight gain or achieving and maintaining modest (5–10% of initial body weight) weight loss) through lifestyle intervention (combination of diet, exercise and behavioural modification) as first line treatment [9]. However weight management is difficult, with attrition a common problem [14] and sustained long-term weight loss a challenge [15]. These outcomes may be potentially worsened in PCOS, with Cochrane reviews reporting attrition rates of up to 46% [16]. Furthermore, clinically significant weight loss is not achieved by all people, with clinical weight loss interventions in the general population reporting 57% of people achieved a ≥5% body weight reduction [17]. Higher attrition and poorer weight management may potentially occur in women with PCOS due to psychosocial (such as the higher prevalence of psychological disorders [8]) or physiological (such as abnormalities in energy expenditure [18] or appetite regulation [19]) factors. 

It would be advantageous to identify factors associated with attrition and weight loss success to guide program development and to identify women pre-treatment who may benefit from increased support or alternative program modalities. Many genetic, physiological, psychosocial and behavioural factors are likely to be involved in attrition and weight loss success in the general population including sociodemographic or psychological factors such as age, education, depression and stress [14]. However, these factors have been poorly studied in PCOS. This is important given PCOS is a prevalent condition with significant obesity-related morbidities in which weight management plays a key role in the treatment. The aim of this study was to identify participant and intervention characteristics associated with attrition and weight loss success in women with PCOS who are overweight or obese participating in weight loss interventions.

## 2. Materials and Methods

### 2.1. Participants

This secondary analysis comprises the combination of four clinical trials assessing the effect of weight loss on anthropometric, reproductive, metabolic or psychological outcomes in women with PCOS (*n* = 221) conducted at the Commonwealth Scientific and Industrial Research Organisation Division of Health Sciences and Nutrition between 2001 and 2007 [1,2,3,4] (Supplementary Table S1). The women were predominantly of European descent (*n* = 218 with *n* = 2 South Asian and *n* = 1 African). 

These studies were combined for assessment of baseline data, changes in anthropometric, reproductive, metabolic and psychological outcomes with weight loss and prediction of intervention attrition and achievement of clinically significant weight loss (≥5%) at 2 months intervention or study completion. The current study included data from all available participants to maximise power. Populations for all studies were (body mass index (BMI) > 25 kg/m^2^) premenopausal women who were overweight or obese, aged 18–45 years. All women had PCOS as classified by the European Society for Human Reproduction and Embryology/American Society for Reproductive Medicine (ESHRE/ASRM) diagnosis [6]. This comprises the presence of two of the three features of hyperandrogenism [either clinical (hirsutism by elevated Ferriman-Gallwey score) or biochemical (elevated testosterone or free androgen index (FAI)), oligo- or amenorrhoea and presence of polycystic ovaries on ultrasound]. 

Study recruitment and inclusion and exclusion have been previously described [1,2,3,4]. Exclusion criteria differed between studies due to the different methodologies and requirements of each individual trial but included weight >140 kg, smoking, type 2 diabetes, pregnancy, breastfeeding, participation in regular physical activity (less than 150 min/week of moderate to vigorous physical activity) and medical (history of cardiovascular, liver, kidney or respiratory disease, diabetes, uncontrolled hypertension or malignancy) or endocrine disorders (congenital adrenal hyperplasia, androgen-secreting tumours, Cushing’s syndrome, hyperprolactinaemia, thyroid dysfunction and adrenal disorders). All participants had ceased oral contraceptives for at least 4 weeks and hormone treatment or insulin-sensitising medications for at least 2 weeks prior to intervention commencement. Eight participants were taking anti-depressant or anti-anxiety medication (*n* = 7 [2] and *n* = 1 [3]). The studies received ethics approval from Commonwealth Scientific and Industrial Research Organisation Division of Health Sciences and Nutrition, The North West Adelaide Health Service, The Royal Adelaide Hospital, the Women’s and Children’s Hospital of South Australia or the University of South Australia and all participants gave written informed consent after full explanation of the purpose and nature of all procedures used. Three of the studies were conducted prior to mandatory clinical trial registration [1,2,3] and the remaining study was registered with the Australian Clinical Trial Registry (ACTRN12606000198527) (4).

### 2.2. Interventions

Intervention duration differed between studies, including studies of 2 months [3], 4 months [1], 5 months [4] and 8 months [2]. All studies assessed body weight at 2 months and this time point was chosen for comparison across the studies for the changes in outcomes. As the additional reproductive, metabolic and psychological outcomes were not consistently measured at 2 months these data are not reported here. The interventions within the studies also differed and included (1) an energy restricted high protein diet (6384.5 ± 1653.1 kJ), the energy restricted high protein diet combined with aerobic exercise or the energy restricted high protein diet combined with aerobic and resistance exercise (*n* = 102) [4]; (2) an energy-restricted diet with two meals replaced daily by commercially available meal replacements (5217.0 ± 772.1 kJ) (Slimfast; Uniliver Australasia, Epping, New South Wales, Australia) (*n* = 27) [3]; (3) an energy-restricted diet with two meals replaced daily by commercially available meal replacements (4904.4 ± 745.2 kJ) (Slimfast; Unilever Australasia, Epping, New South Wales, Australia) followed by a 6 month weight loss maintenance protocol during which participants followed either a carbohydrate-counting or fat counting protocol (5992.3 ± 1873.6 kJ) (*n* = 43) [2]; and (4) a 12 week energy restricted (6296.8 ± 376.3 kJ) and 4 week weight loss maintenance protocol (7609.4 ± 1121.4 kJ) where participants following either a higher or lower protein equicaloric diet (*n* = 49) [1] (Supplementary Table S1). Over the initial 2 months examined for this analysis, 1:1 or group dietitian appointments occurred for all studies fortnightly (week 0, 2, 4, 6) (2-4) or monthly (0, 4) excluding the final appointment [1]. The number of 1:1 or group dietitian appointments differed for each study over the full study duration and ranged from *n* = 4 [1,3] to *n* = 10 [2,4] for the entire study, excluding the final appointment (Supplementary Table S1). 

### 2.3. Clinical and Biochemical Measurements

Following an overnight fast, height and weight (lightly clothed without shoes) were measured and body mass index (BMI) was calculated. BMI was classified as overweight (25.0–29.9 kg/m^2^), obese class I (30.0–34.9 kg/m^2^), obese class II (35.0–39.9 kg/m^2^) and obese class III (≥40 kg/m^2^) according to the World Health Organization classifications [20]. Waist circumference was measured to the nearest 0.5 cm directly on the skin at the level of midway between the lateral lower rib margin and the iliac crest. Fasting venous blood samples were taken for analysis of glucose, insulin, testosterone and sex hormone binding globulin as previously described [1,2,3,4]. Homeostasis assessment of insulin resistance (HOMA-IR) was calculated by [(fasting glucose x insulin)/22.5] [21] and FAI was calculated by [(testosterone/SHBG)] × 100] [22]. Health-related quality of life was assessed by using the validated PCOS quality-of-life questionnaire [23]. Depressive symptoms were assessed as previously described [24] using the Center for Epidemiologic Studies Depression Scale (CES-D) [25], which is a self-reported scale that measures the presence and severity of depressive symptoms in the past week. The scale consists of 20 items with a 4-point rating scale ranging from 0 (rarely, less than 1 day) to 3 (most of the time, 5–7 days) with items on depressed mood, feelings of guilt, worthlessness, helplessness and hopelessness, psychomotor retardation, loss of appetite, and sleep disturbance. Total scores above 16 are considered indicative of the presence of mild depression [26].

### 2.4. Statistics

Two-tailed statistical analysis was performed with Stata (Stata/IC 13.1 for Windows 2014), with statistical significance set at α level of *p* ≤ 0.05. Data were assessed for normality using Kolmorgov-Smirnov tests. Data are presented as mean ±SD (continuous data) and proportions categorical data (categorical data). Analyses were performed comparing women who either did or did not drop out of the studies or who did or did not achieve ≥5% weight loss. Data were analysed using independent *t* tests (continuous data) or chi-square tests (categorical data) for baseline data or repeated measures ANOVA (continuous data) for changes in outcomes over time with dropout or weight loss status as the between subject factor. Univariate or multivariate logistic regression analysis using simultaneous entry of preselected predictors was used to examine demographic, anthropometric and biochemical contributors to the categorical variable dropout or weight loss. The independent variables were selected for each model based on hypothesis testing or associations on correlations (with a *p* value <0.2 considered for inclusion). Regression models were constructed to avoid collinearity with standard model assumptions assessed. Models were also constructed to include the use of medication for depression or anxiety, the study and study duration the participants were involved in and the particular intervention the participants received in that study. These univariate and multivariate prediction models for dropout status or achieving 5% weight loss were conducted using receiver operating characteristic (ROC) curves. The optimal balance between false-positive and false-negative discovery rates was determined by calculating the maximal Youden index across the ROC space.

## 3. Results

Participant characteristics are presented in Table 1. Participants had a mean age of 30.7 ± 6.0 years, weight of 99.9 ± 19.0 kg and BMI of 36.5 ± 6.3 kg/m^2^. 14.4% of the participants were overweight and 85.6% were obese. 

Depressive symptoms scores and presence of mild depression were based on the self-reported Center for Epidemiologic Studies Depression Scale (CES-D). Age is available for *n* = 222 participants due to some participants dropping out of the studies prior to the first appointment.

One hundred and four women dropped out of the weight loss interventions (47.1%) with no differences in attrition rate for each of the 4 substudies (*p* = 0.854). Of these, the majority dropped out prior to 8 weeks (*n* = 77, 74.0%). Of these, *n* = 21 (20.2%) dropped out before study commencement, *n* = 19 (18.3%) dropped out between week 0 and 2, *n* = 8 (7.8%) dropped out between week 2 and 4, *n* = 19 (18.3%) dropped out between week 4 and 6 and *n* = 10 (9.6%) dropped out between week 6 and 8. The remaining *n* = 27 (26.0%) of women dropped out after 2 months. Women who dropped out had lower fasting glucose levels and a better baseline quality of life related to body hair but worse quality of life related to infertility, a trend for elevated scores for depressive symptoms and attended less appointments during the study (either over the initial 2 months or the full study) calculated as % appointments attended both prior to and after dropping out. Participant characteristics are presented in Table 2 according to dropout status. 

Participant characteristics are presented in Table 3 according to weight loss status. Significant changes in weight occurred from baseline to 2 months (5.7 ± 2.9 kg, 5.7 ± 2.6%, *p* < 0.001) or over the entire intervention (7.4 ± 5.3 kg, 7.3 ± 4.9%, *p* < 0.001) for all participants. Of the women who completed 2 months of the intervention or the full interventions, *n* = 85/140 (62.5%) and *n* = 74/118 (62.7%) achieved ≥5% weight loss respectively. Women who achieved ≥5% weight loss over 2 months had better baseline quality of life related to infertility and women who achieved ≥5% weight loss over the full study attended more study appointments.

Multiple logistic regression models were constructed to assess the association between women who did or did not drop out from the studies and participant characteristics (Table 4). Depressive symptoms and lower appointment attendance over the first 2 months of the study were significantly independently associated with being more likely to drop out of the studies. This relationship was maintained on exclusion of the participants taking anti-depressant or anti-anxiety medication. Logistic regression models were then constructed to assess the association between participant characteristics (baseline age, baseline BMI, baseline depressive symptoms and appointment attendance) and not achieving ≥5% weight loss at either 2 months or over the full study duration (Table 4). There were no significant associations between the independent variables and weight loss at 2 months. For the complete study duration, lower appointment attendance was significantly independently associated with not achieving ≥5% weight loss. The ROC curves for the multivariate models for dropouts and not achieving ≥5% weight loss over the entire study produced greater AUC than the univariate models with improved compromises between sensitivity and specificity. All results were maintained on additional assessment of the use of medication for depression or anxiety, the study and study duration the participants were involved in and the particular intervention the participants received in that program.

## 4. Discussion

We report here for the first time that women with PCOS who are overweight or obese with higher levels of depressive symptoms were more likely to drop out of weight loss interventions and that greater appointment attendance may promote less attrition and greater weight loss success. We confirm in the largest sample of women with PCOS studied to date that up to half of women with overweight or obesity and PCOS drop out of clinical research based weight loss interventions [16] but nearly two thirds can lose a clinically significant amount of weight loss once they remain in a professionally supported and structured weight loss program [27]. 

The high level of attrition observed (47%) is consistent with a prior Cochrane review in PCOS [16] and a 9–64% range over 2–4 months in the general population, although only 4/17 of these studies had equivalent or higher attrition rates than we observed here [28]. This supports prior findings of higher attrition rates in identical interventions in populations with and without PCOS (38 vs. 14%) [1,29]. The reason for this potential higher attrition is unclear but may relate to factors such as higher prevalence of psychological disorders [8], abnormalities in energy expenditure [18] or appetite regulation [19] in PCOS. Our finding that 63% of women achieved ≥5% weight loss over 2–8 months is also consistent with prior reports in PCOS [27] and in the general population for clinical research weight loss interventions over 6 months [17]. This highlights that while there is some evidence that women with PCOS may experience equivalent or higher drop-out rates to the general population, significant weight loss can promisingly be achieved once women stay in a structured program. This is consistent with our prior systematic review of equivalent weight loss success for women with and without PCOS in clinical weight management programs [30].

This is the first report in PCOS showing that higher appointment attendance was associated with both reduced attrition and achieving clinically significant weight loss. This is consistent with prior research in the general population reporting appointment attendance or treatment time as independent predictors of attrition or weight loss success [17,31]. Appointment attendance may also be a surrogate measure of adherence or compliance although here we lack detailed data on exercise or dietary compliance. While there was a marked difference in appointment attendance for women who did and did not drop out of the study (60 vs. 96%), this was more subtle for those who did and didn’t achieve significant weight loss (84 vs. 94%). This is to be expected as even when women are committed to staying in a lifestyle intervention, attending each appointment is critically important and impacts on the likelihood of successful weight loss. Lack of attendance may also be a marker that could signal the need for improved participant support. These points should be communicated to participants and study investigators. However, we need to recognise the burden given that multifactorial lifestyle interventions can have up to 26 appointments/12 months when models such as the Diabetes Prevention Program are used as an exemplar [32]. The use of alternative and remote delivery modes should be considered such as computer and mobile phone based interventions which still have a degree, albeit lesser, of success [33,34]. These may also be more appealing to younger populations [35], such as premenopausal women with PCOS included here or young women without PCOS studied elsewhere [36], who have greater personal commitments and less time availability [37] and have lower adherence, higher attrition or lower weight loss in standard weight management interventions [17]. 

We report for the first time that a higher level of depressive symptoms was associated with greater program attrition in PCOS. This is consistent with research in the general population [31,38,39] and in young women without PCOS [36] which may be related to factors such as poor self-esteem and self-efficacy. This highlights the importance of assessing psychological outcomes in PCOS for identifying women to offer greater psychological support for improving adoption and sustaining of healthy lifestyle behaviours and weight management. This is consistent with similar or greater weight loss for individuals with depression in the general population undertaking a structured clinical weight management program when offered additional psychological input [40]. This is particularly important given weight loss also improves psychological outcomes in PCOS [24]. In keeping with this, the use of allied health referrals (including psychology in addition to dietetics and exercise physiology) is recommended to support medical weight management [41] and highlighted in both evidence-based guidelines for PCOS [9] and the general population [42,43,44]. However, the actual use of allied health clinicians for supporting weight management is unclear. Fewer than 1/1000 Australian women by 27 years used subsidised allied health (dietetic, exercise physiology or psychology) referrals for chronic disease management which increases to 108/1000 by 42 years [41]. Further research is needed to understand how to support a multidisciplinary approach in the management of overweight and obesity in PCOS. In contrast to general population research [31], in the present study depressive symptoms were not associated with achieving clinically significant weight loss. This may indicate that once women with PCOS are committed to a lifestyle intervention that this can be sustained despite any underlying psychological disturbance. Alternatively, as women with a more severe level of depression may be more likely to drop out of a study, this may then reduce the possibility of observing a significant relationship with weight loss success at a later stage in the study.

There was no relationship observed between demographic, anthropometric, clinical or hormonal factors with attrition or weight loss success. This contrasts with prior studies in the general population where factors such as age and BMI have been associated with attrition or achieving clinically significant weight loss [14,28,31,45]. The exact reasons for these differences is unclear, but underlying issues such as a higher prevalence of depression in PCOS [8] may outweigh other subtle associations. Of note, we report here worsened baseline quality of life related to infertility was related to higher attrition and a lower proportion of achieving ≥5% weight loss over the first 2 months of the intervention. For women with worse quality of life related to infertility, conceiving may be a higher concern for them for which they may wish to seek forms of treatment for which they see a more immediate benefit than committing the effort to achieving weight change [46]. Given that weight loss can improve the outcome of medical infertility treatment [47,48], the importance of lifestyle management concurrently with infertility treatment may be a clinical message that warrants greater effort to be communicated to and taken up by patients. 

Limitations of this study include heterogeneity across intervention characteristics including duration, intensity, modality and study measurements which impacted our ability to combine findings. However, the lack of effect of intervention (use of or type of diet and exercise) supports our finding that it is overall compliance rather than modality that is associated with weight loss success. This is consistent with our prior reports of similar results with varying diet compositions [49] and physical activity [50] interventions in PCOS and evidence-based guidelines recommendations that there is no one optimal diet or physical activity approach in PCOS for weight management and that general population guidelines should be followed [9]. This is also consistent with similar findings in the general population where dietary adherence had a stronger effect on long-term weight loss than diet composition [51]. We were not able to investigate the full range of potential factors associated with study attrition or weight loss success such as weight loss expectations, self-efficacy, motivation, ethnicity, education, employment status, socioeconomic status, smoking, social support and chronic diseases [14,28,31,45,52] due to the predefined nature of our populations and study measurements. These should be investigated further in PCOS. As the study population was predominantly of a European descent, the findings of the current analysis cannot be directly extrapolated for women of different ethnic backgrounds. This was a controlled clinical research study and as such the generalisability to a free-living environment is limited. However, prior research has reported women with PCOS in a regular medical practice can also achieve clinically meaningful weight loss, albeit to a lesser degree than that achieved here (32% over 2–6 months) [53]. The research studies also lacked a structured behavioural component and focused solely on dietary or exercise changes. While our data does not show cause and effect, the inclusion of a behavioural component, as recommended in evidence-based guidelines for PCOS [9] and the general population [42,43,44], would possibly reduce attrition and improve weight loss success further. It may also provide greater support for women with underlying psychological disturbances as present in PCOS. This warrants further assessment. We also note that this study is an exploratory analysis only as these hypotheses were not developed a priori and there was no adjustment for multiple testing.

## 5. Conclusions

In conclusion, we report in the largest study to date that while attrition may affect up to half of women with PCOS undergoing professionally supervised and structured weight loss interventions in a clinical research setting, clinically meaningful weight loss can be achieved by up to two thirds of women who remain in these types of interventions. We also report for the first time in PCOS that women with higher levels of depressive symptoms have greater attrition rates and that appointment attendance is associated with improved attrition and weight loss success. This has important implications for the appropriate screening and support of women with PCOS requiring weight loss interventions.

## Figures and Tables

**Table 1:** Characteristics of study participants.

Outcome	N	Mean ± SD
Age (years)	221	30.7 ± 6.0
Weight (kg)	201	99.8 ± 19.0
BMI (kg/m^2^)	201	36.5 ± 6.3
BMI (categories)	201	Overweight: 29 (14.4%)
		Obese: 172 (85.6%)
		Obese class I: 66 (32.8%)
		Obese class II: 53 (26.4%)
		Obese class III: 53 (26.4%)
Waist circumference (cm)	155	103.5 ± 13.2
Fasting insulin (mU/L)	186	16.9 ± 10.5
Fasting glucose (mmol/L)	204	5.2 ± 0.6
HOMA-IR	186	4.1 ± 2.8
Testosterone (nmol/L)	204	2.4 ± 0.9
SHBG (nmol/L)	204	29.6 ± 15.4
FAI	204	11.5 ± 13.1
FG	177	15.9 ± 8.3
QOL emotional state	136	4.4 ± 1.1
QOL body hair	136	3.6 ± 1.5
QOL weight status	136	2.2 ± 1.0
QOL infertility	136	4.5 ± 1.7
QOL menstrual irregularity	136	3.6 ± 1.2
Depressive symptoms	93	18.3 ± 9.9
Presence of mild depression	93	Yes: 49 (52.7%)
		No: 44 (47.3%)

Data is presented as mean ± SD or n (proportions) BMI: body mass index; FAI: free androgen index; FG: ferriman-gallwey score; HOMA-IR: homeostasis assessment of insulin resistance; QOL: quality of life; SHBG: sex hormone binding globulin.

**Table 2:** Characteristics of study participants according to dropout status.

Outcome	Dropouts *N* = 104	Non-Dropouts *N* = 117	MD, 95% CI, *p*
Age (years)	30.0 ± 6.1	31.4 ± 5.7	1.32 (−0.26, 2.90), *p* = 0.101
Weight (kg)	100.0 ± 17.8	99.7 ± 19.9	−0.33 (−5.69, 5.03), *p* = 0.903
BMI (kg/m^2^)	36.5 ± 6.4	36.4 ± 6.2	−0.08 (−1.85, 1.69), p = 0.933
BMI (categories)	Overweight (13.1%) Obese (86.9%)	Overweight (15.4%) Obese (84.6%)	*p* = 0.649
Waist circumference (cm)	102.1 ± 12.0	104.7 ± 14.0	2.53 (−1.70, 6.77), *p* = 0.239
Fasting insulin (mU/L)	16.0 ± 10.4	17.4 ± 10.6	1.43 (−1.72, 4.57), *p* = 0.373
Fasting glucose (mmol/L)	5.1 ± 0.49	5.3 ± 0.63	0.19 (0.03, 0.35), *p* = 0.019
HOMA-IR	3.7 ± 2.6	4.2 ± 2.9	0.51 (−0.33, 1.36), *p* = 0.235
Testosterone (nmol/L)	2.4 ± 0.89	2.5 ± 0.93	0.06 (−0.19, 0.32), *p* = 0.627
SHBG (nmol/L)	30.9 ± 17.2	28.6 ± 13.9	−2.35 (−6.65, 1.96), *p* = 0.284
FAI	10.0 ± 6.5	12.6 ± 16.3	2.60 (−1.04, 6.25), *p* = 0.161
FG	14.8 ± 8.4	16.9 ± 8.1	2.13 (−0.33, 4.59), *p* = 0.089
QOL emotional state	4.3 ± 1.1	4.5 ± 1.1	0.23 (−0.16, 0.61), *p* = 0.242
QOL body hair	3.9 ± 1.6	3.4 ± 1.3	−0.56 (−1.05, −0.07), *p* = 0.026
QOL weight status	2.1 ± 1.0	2.3 ± 1.1	0.20 (−0.15, 0.55), *p* = 0.264
QOL infertility	4.1 ± 1.8	4.8 ±1.5	0.62 (0.06, 1.18), *p* = 0.030
QOL menstrual irregularity	3.6 ± 1.3	3.6 ± 1.1	0.02 (−0.38, 0.43), *p* = 0.906
Depressive symptoms	20.5 ± 11.2	16.5 ± 8.4	−4.02 (−8.06, 0.02), *p* = 0.051
Presence of mild depression	57.1%	49.0%	*p* = 0.435
Appointment attendance—2 months (%)	59.6 ± 39.6	96.2 ± 14.9	*p* < 0.001
Appointment attendance—full study weeks (%)	24.1 ± 24.8%	90.1 ± 18.7%	*p* < 0.001
Appointment attendance—2 months (categories)	75–100%: 53.9% <75%: 46.2%	75–100%: 97.4% <75%: 2.6%	*p* < 0.001
Appointment attendance—full study (categories)	75–100%: 5.8% <75%: 94.2%	75–100%: 85.5% <75%: 14.5%	*p* < 0.001

Data is presented as mean ± SD or proportions and were analysed as paired *t*-test (baseline values) or chi squared test (proportions) with dropout status as the between subject factor. BMI: Body mass index; CI: Confidence interval; FAI: Free androgen index; FG: Ferriman-gallwey score; HOMA-IR: homeostasis assessment of insulin resistance; MD: Mean difference; QOL: Quality of life; SHBG: Sex hormone binding globulin.

**Table 3:** Characteristics of study participants according to weight loss status.

	2 Months			Full Study Duration		
Outcome	<5% weight loss *N* = 55	≥5% weight loss *N* = 85	MD, 95% CI, *p*	<5% weight loss *N* = 44	≥5% weight loss *N* = 74	MD, 95% CI, *p*
**Age (years)**	31.7 ± 5.9	31.2 ± 5.5	−0.53 (−2.51, 1.44), *p* = 0.594	32.5±5.5	30.6±5.8	-1.88 (-4.03, -0.27), *p* = 0.09
**Weight (kg)**	98.3 ± 17.7	99.3 ± 20.3	0.96 (−5.84, 7.76), *p* = 0.780	97.1±19.3	101.0±20.1	3.93 (-3.54, 11.40), *p* = 0.300
**BMI (kg/m^2^)**	36.9 ± 6.0	35.9 ± 6.3	−1.03 (−3.20, 1.14), *p* = 0.348	35.9±6.1	36.7±6.2	0.77 (-1.57, 3.10), *p* = 0.517
**BMI (WHO categories)**	Overweight 11.8% Obese 88.2%	Overweight 18.8% Obese 81.2%	*p* = 0.279	Overweight 20.5% Obese 79.6%	Overweight 13.5% Obese 86.5%	*p* = 0.321
**Waist circumference (cm)**	103.9 ± 11.9	103.6 ± 15.0	−0.23 (−5.74, 5.27), *p* = 0.933	105.0±12.5	104.4±15.2	-0.57 (-6.56, 5.42), *p* = 0.851
**Fasting insulin (mU/L)**	16.8 ± 10.4	16.6 ± 10.1	−0.16 (−3.78, 3.45), *p* = 0.929	17.2±10.9	17.5±10.4	0.22 (-3.77, 4.21), *p* = 0.914
**Fasting glucose (mmol/L)**	5.3 ± 0.65	5.3 ± 0.57	0.01 (−0.20, 0.22), *p* = 0.898	5.3±0.5	5.3±0.7	-0.002 (-0.24, 0.24), *p* = 0.988
**HOMA-IR**	4.0 ± 2.6	4.1 ± 3.0	0.07 (−0.93, 1.07), *p* = 0.894	4.2±2.8	4.3±3.0	0.07 (-1.04, 1.19), *p* = 0.895
**Testosterone (nmol/L)**	2.6 ± 1.0	2.4 ± 0.92	−0.13 (−0.47, 0.20), *p* = 0.432	2.6±0.84	2.4±0.96	-0.25 (-0.59, 0.10), *p* = 0.163
**SHBG (nmol/L)**	29.2 ± 13.2	28.5 ± 14.4	−0.68 (−5.57, 4.22), *p* = 0.784	28.3±14.4	28.7±13.7	0.42 (-4.84, 5.67), *p* = 0.874
**FAI**	12.1 ± 12.2	12.7 ± 17.1	0.63 (−4.79, 6.04), *p* = 0.819	13.2±13.6	12.2±17.7	-0.95 (-7.09, 5.19), *p* = 0.760
		**2 months**			**Full study duration**	
**FG**	16.4 ± 9.0	16.5 ± 7.9	0.08 (−3.13, 3.28), *p* = 0.962	15.8 ± 8.2	17.6 ± 8.0	1.75 (−1.65, 5.16), *p* = 0.309
**QOL emotional state**	4.4 ± 1.2	4.5 ± 1.0	0.02 (−0.46, 0.51), *p* = 0.920	4.5 ± 1.0	4.5 ± 1.2	0.03 (−0.50, 0.57), *p* = 0.899
**QOL body hair**	3.6 ± 1.4	3.5 ± 1.5	−0.12 (−0.76, 0.52), *p* = 0.708	3.4 ± 1.3	3.3 ± 1.4	−0.13 (−0.77, 0.51), *p* = 0.683
**QOL weight status**	2.0 ± 0.9	2.4 ± 1.1	0.44 (−0.02, 0.91), *p* = 0.061	2.3 ± 1.1	2.3 ± 1.0	−0.05(−0.55, 0.46), *p* = 0.857
**QOL infertility**	4.3 ± 1.4	5.1 ± 1.5	0.76 (0.13, 1.40), *p* = 0.02	4.7 ± 1.5	4.8 ± 1.6	0.13 (−0.60, 0.86), *p* = 0.731
**QOL menstrual irregularity**	3.7 ± 1.0	3.6 ± 1.1	−0.09 (−0.56, 0.39), *p* = 0.720	3.7 ± 1.2	3.6 ± 1.1	−0.13 (−0.66, 0.41), *p* = 0.640
**Depressive symptoms**	15.6 ± 7.6	17.8 ± 9.1	2.13 (−2.57, 6.83), *p* = 0.368	14.3 ± 5.3	17.6 ± 9.4	3.32 (−1.74, 8.38), *p* = 0.193
**Presence of depression**	45.5%	55.9%	*p* = 0.446	37.5%	54.3%	*p* = 0.266
**Appointment attendance (%)**	96.1 ± 10.5	97.1 ± 15.6	0.98 (−3.90, 5.86), *p* = 0.692	84.0 ± 22.4	93.9 ± 15.0	9.87 (3.06, 16.69), *p* = 0.005
**Appointment attendance (%)-categories**	75–100%: 98.0% <75%: 2.0%	75–100%: 91.7% <75%: 8.3%	*p* = 0.880	75–100%: 72.7% <75%: 27.3%%	75–100%:93.2% <75%: 6.9%	*p* = 0.002

Data is presented as mean ± SD or n (proportions) and were analysed as paired *t*-test (baseline values) or chi squared test (proportions) with weight loss status as the between subject factor. BMI: Body mass index; CI: Confidence interval; FAI: Free androgen index; FG: Ferriman-gallwey score; HOMA-IR: Homeostasis assessment of insulin resistance; MD: Mean difference; QOL: Quality of life; SHBG: Sex hormone binding globulin.

**Table 4:** Multivariate logistic regression models and receiver operator characteristics curves assessing the association of baseline participant characteristics with dropout status and achieving 5% weight loss.

	Age	BMI	Depressive Symptoms	Attendance	Full Model
**Dropouts**					
OR (95% CI), P	0.96 (0.89, 1.05) *p* = 0.406	1.01 (0.92, 1.11) *p* = 0.800	1.07 (0.88, 0.96) *p* = 0.032	0.92 (0.88, 0.96) *p* < 0.001	-
ROC AUC, 95% CI	0.57 (0.49, 0.64)	0.50 (0.41, 0.58)	0.60 (0.48, 0.72)	0.77 (0.72, 0.83)	0.82 (0.72, 0.91)
ROC optimal sensitivity (%), optimal specificity (%)	0.49, 0.67	0.07, 0.99	0.33, 0.90	0.62, 0.91	0.79, 0.75
**<5% weight loss 2 months**					
OR (95% CI), P	1.05 (0.96, 1.15) *p* = 0.313	0.94 (0.85, 1.05) *p* = 0.276	0.97 (0.91, 1.04) *p* = 0.406	0.94 (0.87, 1.01) *p* = 0.088	-
ROC AUC, 95% CI	0.54 (0.43, 0.64)	0.55 (0.45, 0.65)	0.56 (0.41, 0.71)	0.54 (0.49, 0.60)	0.71 (0.57, 0.84)
ROC optimal sensitivity (%), optimal specificity (%)	0.59, 0.54	0.84, 0.57	0.62, 0.58	0.14, 0.95	0.77, 0.56
**<5% weight loss full study**					
OR (95% CI), P	1.06 (0.95, 1.18) *p* = 0.298	1.01 (0.90, 1.13) *p* = 0.845	0.96 (0.88, 1.04) *p* = 0.335	0.95 (0.92, 0.99) *p* = 0.020	-
ROC AUC, 95% CI	0.59 (0.48, 0.70)	0.53 (0.42, 0.64)	0.60 (0.44, 0.75)	0.64 (0.54, 0.73)	0.75 (0.60, 0.90)
ROC optimal sensitivity (%), optimal specificity (%)	0.55, 0.68	0.32, 0.80	0.94, 0.37	0.50, 0.73	0.75, 0.74

AUC: Area under the curve; BMI: body mass index; ROC: Receiver operator characteristics curve Data are presented as OR, 95% CI or AUC and were analysed by logistic regression or by ROC curve.

## Supplementary Materials

The following are available online at https://www.mdpi.com/2072-6643/11/3/492/s1, Table S1: Intervention characteristics of included studies.
