# Supplementary material for: Predictors of Lifestyle Intervention Attrition or Weight Loss Success in Women with Polycystic Ovary Syndrome Who Are Overweight or Obese
Source: Nutrients. 2019 Feb 26;11(3):492. doi: 10.3390/nu11030492 (PMC6470873; doi:10.3390/nu11030492)
Supplement: Supplementary file 1 [file nutrients-11-00492-s001.pdf]

Supplementary Table 1: Intervention characteristics of included studies

Author	Year	n	Intervention type	Intervention duration	Time points	Appointment number	Appointment type	Attrition	Weight loss
Moran	2003	Commenced: 49<br>Randomised: 44 (22 LP, 22 HP)<br>Completed: 28 (14 LP 14 HP)	Weight loss with HP (40% CHO, 30% P, 30% F) or LP (55% CHO, 15% P, 30% F) diet and weekly exercise class	12 week weight loss<br>4 week weight maintenance	0, 4, 8, 12, 16	5	1:1 dietitian	42.9%	7.7±0.7 kg<br>NS between groups
Moran	2006	Commenced: 43 (22 CC, 21 FC)<br>Completed: 23 (14 CC, 9 FC)	Meal replacements for weight loss for all participants followed by CC or FC for weight maintenance	8 week weight loss<br>6 month weight maintenance	0, 2, 4, 6, 8, 12, 16, 20, 24, 28, 32	11	1:1 dietitian	46.5%	4.7±1.0 kg<br>NS between groups
Moran	2007	Commenced: 27<br>Completed: 15	Meal replacements for weight loss	8 week	0, 2, 4, 6, 8	5	1:1 dietitian	44.4%	3.9±3.6 kg
Thomson	2008	Commenced: 102<br>Randomised: 100 (32 HP, 34 HP/AT, 34 HP/ART)<br>Completed: 51 (14 HP, 18 HP/AT, 20 HP/ART	HP, HP/AT or HP/ART for weight loss	20 week	0, 2, 4, 6, 8, 10, 12, 14, 16, 18, 20	11	1:1 or group dietitian and exercise physiologist sessions	50.0%	9.4±1.9%<br>NS between groups

ART: aerobic resistance training; AT: aerobic training; CC: carbohydrate counting, CHO: carbohydrate; F: fat; FC: fat counting, HP: high protein; LP: low protein; NS: not significant; P: protein
